# Remarks on the Exhibition of Sulphate of Quinine in Acute Rheumatism

**Published:** 1830-10-01

**Authors:** 


### SULPHATE OF QUININE IN ACUTE RHEUMATISM.

OUR readers know that Dr. Haygarth, and many others since his time, ventured to treat acute rheumatism—in common parlance, rheumatic fever, by bark instead of depletion. Numerous cures, and speedy ones too, are reported in the periodical journals, on this plan; and these reports, even admitting them "cum grano salis," as medical reports should generally be received, prove sufficiently that rheumatic is not common inflammation. The intensely buffed state of the blood, however, the frequent metastasis of the phlogosis to the heart, the colour of the urine, the temperature of the skin, the thirst, and the rapidity of the circulation, should induce us to be cautious how we administer powerful tonics in such conditions of the system. We have been led to these preliminary remarks by the hospital-report of a case from the wards of the Royal Infirmary of Edinburgh, recently published; and as the case is very short, we shall first introduce it to our readers.

"Catherine Morgan, ætat. 30, admitted on the fifth day of fever, accompanied with symptoms of acute rheumatism; *she had likewise cough, diffused pain in the chest, increased by full inspiration; her respiration was hur-*

*ried; expectoration mucous and adhesive; pulse frequent;* bowels slow; she had a similar attack two months before. On admission, the symptoms of rheumatism were so prominent, as *to hide or obscure many of the others.* It was to this affection, consequently, that Dr. Duncan's attention was especially attracted; indeed the pectoral complaints were scarcely noticed at all till the day after he had ordered the remedy he considered it expedient to use against the rheumatism, this was the sulphate of quinine, concerning the efficacy of which, even in the most acute form of this disease, he had already given so decided an opinion; in this case, however, its administration did mischief to a certain extent, for on the following morning the cough and dyspnœa could no longer be overlooked, and some annoying abdominal symptoms also made their appearance, the epigastrium being very tender to the touch, with considerable internal pain. The quinine was of course immediately omitted, and leeches were applied; their operation was followed with great relief, and during the night she slept better, and perspired abundantly. After this, the treatment of the case was exclusively antiphlogistic, and she was dismissed cured on the 29th of March."

How the cough, pain of chest, hurried respiration, quick pulse, and tenacious expectoration could be overlooked, after being noted on the day of admission—and especially by so able an observer as Dr. Duncan, we are unable to even imagine. It is distinctly stated that these symptoms *accompanied* the symptoms of *acute rheumatism* on admission—and yet the latter did *hide or obscure* the former!! We are very much inclined to suspect that these thoracic symptoms were occasioned by the administration of the tonic, as well as the pain and tenderness of the epigastrium. Such cases as these give fair scope for scandalization among our Broussaian brethren of the continent, and the ignorant or careless—perhaps mendacious manner of reporting them increase the evil tenfold. It is therefore necessary that a check should go forth with the evil, to show that we are not blind empirics and indiscriminate routinists on this side of the channel. We are disposed to think that the mercurial practice in acute rheumatic inflammation, adopted by Dr. Chambers and many other observant practitioners on this side of the Tweed, is far preferable to the tonic and stimulant treatment recorded in the foregoing case.

The same accurate and veracious reporter tells us something about a case of "articular rheumatism" which yielded to the antiphlogistic treatment, "followed up by the *use* of colchicum wine, in the dose of *three grains*, repeated thrice daily." Verily the reporter and his editorial master are clever directors of the practice of medicine in this country!!

In the same luminous report, we are informed that a young woman, who was evidently labouring under one of the anomalous forms of hysteria, (which in this case, assumed the character of Nephritis) was bled to 163—but whether ounces or pounds the reporter saith not. If any confidence indeed could be placed in the report, we should be rather surprized that the talented Dr. Duncan did not, at once, attribute the complaint to its proper class, and spare the effusion of human blood. After repeated leechings and other depletive measures, "she was suddenly attacked with a similar pain (to that in the region of the kidneys) under the sternum." Although the hysterical Proteus shifted his ground, he did not elude the persevering enemy who depleted to 163 ounces or pounds!

